# S122. 3-YEAR NEGATIVE SYMPTOM TRAJECTORY AND ITS RELATIONSHIP WITH SYMPTOM AND FUNCTIONAL OUTCOMES IN FIRST-EPISODE NON-AFFECTIVE PSYCHOSIS: A PROSPECTIVE 13-YEAR FOLLOW-UP STUDY

**DOI:** 10.1093/schbul/sby018.909

**Published:** 2018-04-01

**Authors:** Wui Hang Ho, Wing Chung Chang, Yee Man Tang, Lai Ming Hui, Kit Wa Chan, Ho Ming Lee, Yi Nam Suen, Eric Chen

**Affiliations:** The University of Hong Kong

## Abstract

**Background:**

Negative symptoms are a core feature of schizophrenia and are a major determinant of functional impairment. Few studies have been conducted to examine patterns of longitudinal course of negative symptoms in the early stage of illness. Differential relationships of negative symptom trajectories with long-term clinical and functional outcomes remain to be clarified. This study aimed to investigate patterns of negative symptom trajectories over 3 years, utilizing latent class growth analysis (LCGA), in patients presenting with first-episode non-affective psychosis. Predictive capacity of symptom trajectories on 13-year functional and negative symptom outcomes was also examined.

**Methods:**

One hundred thirty-six Chinese patients aged 18–55 years presenting with DSM-IV first-episode schizophrenia, schizoaffective disorder, schizophreniform disorder, brief psychotic disorder or delusional disorder were assessed at clinical stabilization for first psychotic episode (baseline), 1, 2, 3 and 13 years of follow-up. Assessments encompassing premorbid adjustment, baseline symptom and cognitive profiles and functional levels were conducted. Negative symptoms were measured by High Royds Evaluation of Negativity (HEN) Scale. Individual class membership of negative symptoms derived from LCGA was based on HEN ratings at baseline, 1, 2 and 3-year follow-up.

**Results:**

Three distinct negative symptom trajectories were identified including low-stable (59.6%, n=81), moderate-stable (29.4%, n=40) and high-increasing (11.0%, n=15) trajectories. Multinomial regression analysis revealed that poorer premorbid adjustment, lower baseline cognitive composite scores and more severe baseline depression predicted high-increasing trajectory membership (Nagelkerke pseudo R2=0.339, Model χ2=277.96, p<0.01). At 13 years, 88 patients (64.7%) completed follow-up assessment, with attrition analysis indicating lack of significant differences in demographic, premorbid and baseline characteristics between completers and non-completers. Analysis of covariance (controlling for premorbid adjustment, baseline cognition and depression) followed by post-hoc comparison analyses found that high-increasing trajectory was significantly associated with poorer global functional outcome and higher negative symptom levels at 13-year follow-up.

**Discussion:**

Our results indicate that 11% of first-episode non-affective psychosis patients displayed persistently high levels of negative symptoms with gradual symptom worsening over 3-year follow-up. This trajectory membership was predictive of poorer negative symptom and functional outcomes 13 years after presentation. High-increasing negative symptom trajectory identified in the initial 3 years of treatment for first-episode psychosis may represent a subgroup of patients having markedly elevated risk of developing deficit syndrome in the later course of illness.

